# Acupuncture for erysipelas of lower limbs: A case report

**DOI:** 10.1097/MD.0000000000042413

**Published:** 2025-05-09

**Authors:** Bangwei Li, Xuanxuan Ren

**Affiliations:** a Department of Acupuncture and Moxibustion, The Third Affiliated Hospital of Zhejiang Chinese Medical University, Hangzhou, China; b Department of Traditional Chinese Medicine, Zhejiang Hospital, Hangzhou, China.

**Keywords:** acupuncture, case report, erysipelas of lower limbs

## Abstract

**Rationale:**

Erysipelas of the lower limbs is a common acute infectious disease, with clinical manifestations of local skin redness, swelling and pain in the lower limbs, and reports of the use of acupuncture alone for treatment are rare. We report a case of erysipelas of lower limbs that was successfully treated with acupuncture.

**Patient concerns:**

A 31-year-old male patient presented with pain in the right ankle, along with redness and swelling on the inner aspect of the right calf. Physical examination revealed erythema and swelling of the skin near the ankle. The skin temperature was slightly elevated and the patient experienced localized tenderness. In addition, the patient reported severe pain in the right ankle joint while standing upright. The patient had a history of tinea pedis with no evidence of insect bites or trauma. Local acupuncture treatment was administered, and after 3 sessions, the pain resolved, the swelling reduced, and the body temperature normalized.

**Diagnoses:**

We diagnosed the patient with erysipelas of the lower limb.

**Interventions:**

An acupuncture treatment plan was developed based on the patient’s symptoms and diagnosis.

**Outcomes:**

Acupuncture significantly improved pain and swelling associated with erysipelas of the lower limbs.

**Lessons:**

Following acupuncture treatment, the patient’s pain and local redness disappeared, suggesting that acupuncture may be effective in treating erysipelas of the lower limbs. However, owing to the limitations of this single, uncontrolled case study, definitive conclusions cannot be drawn. Further research into the use of acupuncture for treating lower limb erysipelas is warranted in the future. In addition, it is recommended that the patient continue standard treatment for tinea pedis to prevent the recurrence of lower limb erysipelas.

## 1. Introduction

Erysipelas is an acute inflammation of the skin and reticular lymphatic vessels caused by group A β-hemolytic streptococci (*Streptococcus pyogenes*), typically occurring in the lower limbs and face.^[1]^ Antibiotics, such as penicillin, are the first-line treatment. Although antibiotics play a significant role in controlling inflammatory reactions, the potential for organism resistance due to prolonged use cannot be ignored.^[2]^ A comprehensive literature review revealed few reports of on the treatment of lower-extremity erysipelas with acupuncture alone. Here, we present a case of lower limb erysipelas successfully treated with acupuncture, suggesting acupuncture as a potential adjunctive treatment option.

## 2. Case presentation

### 2.1. Case

**Figure 1.**
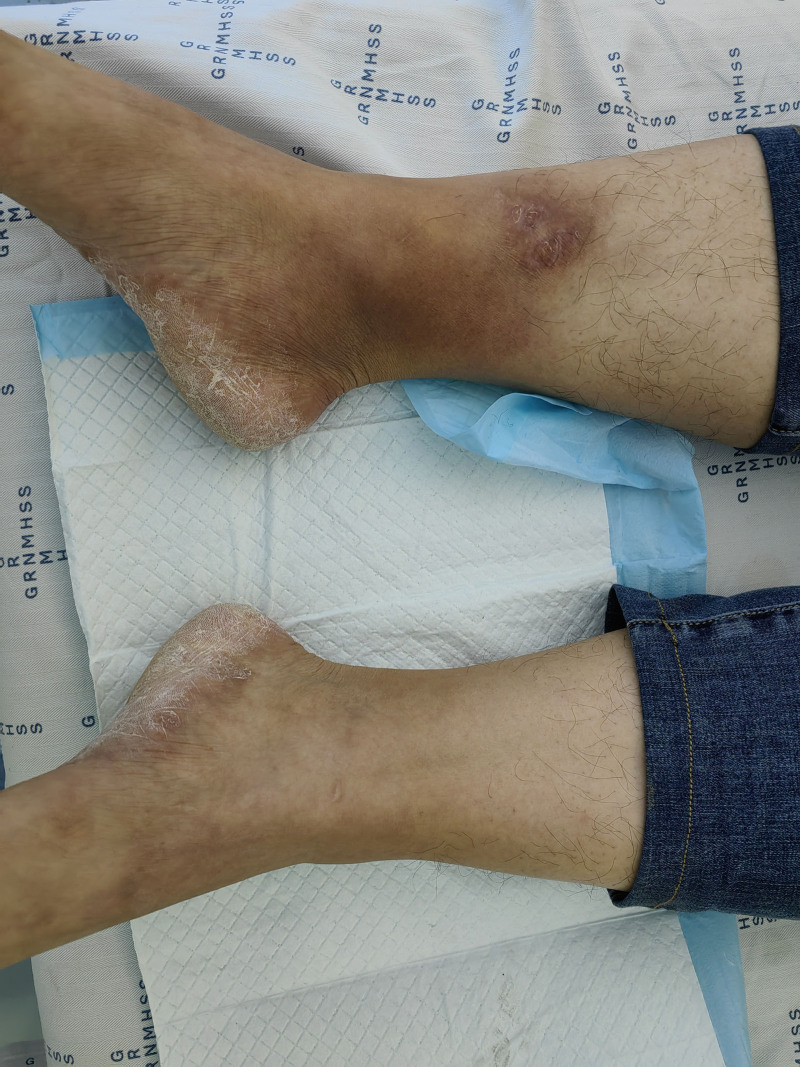
Skin condition at the time of presentation.

A 31-year-old male patient presented with a complaint of “right ankle pain and redness and swelling on the inside of the right calf for 3 days.” On June 14, 2024, the patient developed right ankle pain and was unable to walk or bear weight after sitting for a prolonged period of time. The right calf exhibited erythema and edema accompanied by elevated local skin temperature. He underwent a lower-extremity venous B-ultrasound examination at a local hospital, which did not reveal any evidence of venous thrombosis. No drugs or other treatment was administered. On June 17, 2024, the patient was admitted to the Chinese Acupuncture Department of Katutura Hospital in Windhoek, Namibia, where a Chinese medical team attended. During the physical examination at admission, redness and swelling of the skin were observed on the right inner calf near the inner ankle (Fig. 1). The skin temperature was slightly elevated, and the patient experienced localized pressure pain. Additionally, the patient reported severe pain in the right ankle joint while standing upright. An old scar on the inner side of the right calf, caused by trauma approximately 3 years ago, was noted with no significant discomfort at present. The patient had a history of untreated tinea pedis for 1 year, evidenced by skin flaking on both heels without any other discomfort. There was no evidence of insect bites or recent trauma. Therefore, we diagnosed the patient with erysipelas of lower limb. The treatment of this patient has been approved by the patient’s informed consent, and a written informed consent was obtained from the patient for the release of this case.

### 2.2. Treatment and intervention

**Figure 2.**
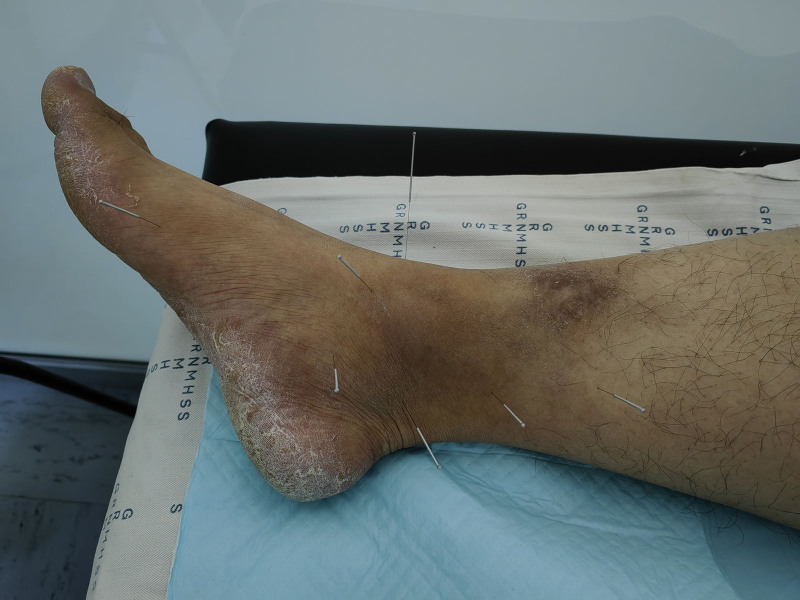
Acupuncture point selection and operation.

An acupuncture treatment plan was developed based on the patient’s symptoms and diagnosis. The primary observation index was the visual analog scale (VAS) score, Which is commonly used for clinical pain assessment. The patient’s VAS score was recorded at 8 points before acupuncture treatment. Acupuncture points were primarily selected around the right ankle joint (Fig. 2), including the Ashi point, Jiexi (stomach meridian of foot-yangming [ST 41]), Taixi (kidney meridian of foot-shaoyin [KI 3]), Zhaohai (KI 6), Gongsun (spleen meridian of foot-taiyin [SP 4]), Shangqiu (SP 5), and Sanyinjiao (SP 6), according to the World Health Organization’s criteria for acupoint locations (Table 1). After skin disinfection, 0.30 × 40 mm acupuncture needles were inserted into the acupoints. All needles were stimulated to achieve a de qi sensation and retained for 20 minutes. Notably after needle removal, there was no need to press the insertion site. Minimal bleeding, if any, was allowed to subside. The treatment was administered once every other day for a total of 3 sessions. After each session, the patient reported pain relief and reduced in skin discoloration.

**Table 1:** Location of acupoints.

Acupoints	Location
Ashi point	Ashi point has not definite location and it is mainly located in the most painful site or trigger point
Jiexi (ST 41)	In the depression at the midpoint of the transverse crease of the ankle between the tendons of m. extensor hallucis longus and digitorum longus
Taixi (KI 3)	Posterior to the medial malleolus, in the depression between the tip of the medial malleolus and tendo calcaneus
Zhaohai (KI 6)	In the depression below the tip of the medial malleolus
Gongsun (SP 4)	On the medial aspect of the foot, in the depression distal and inferior to the base of the first metatarsal bone
Shangqiu (SP 5)	In the depression distal and inferior to the medial malleolus, midpoint between the tuberosity of the navicular bone and the tip of the medial malleolus
Sanyinjiao (SP 6)	3 cun above the medial malleolus, on the posterior border of the medial aspect of tibia

### 2.3. Clinical outcome

**Figure 3.**
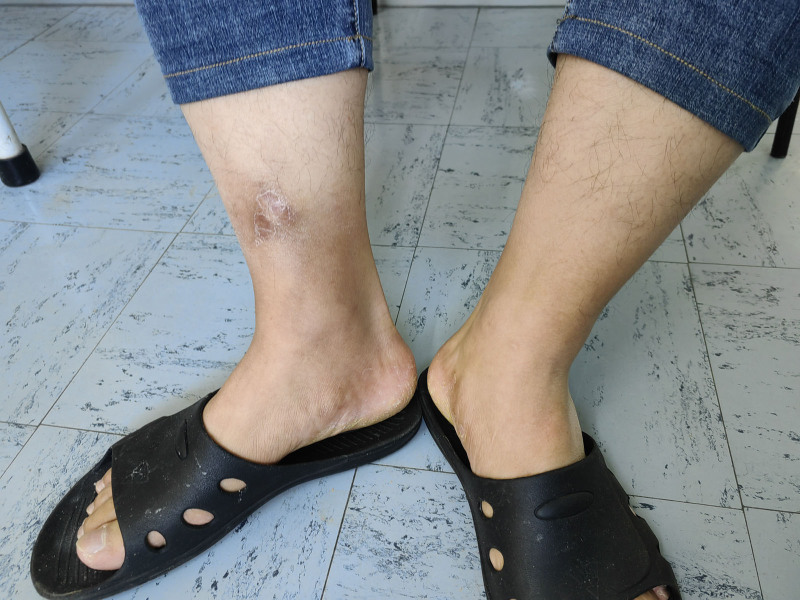
Skin condition after 3 acupuncture treatments.

**Figure 4.**
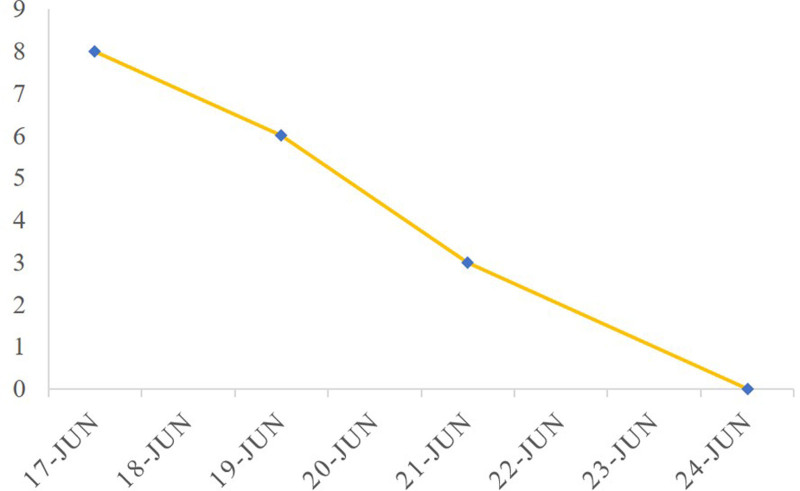
VAS. VAS = visual analog scale.

On June 24, 2024, after 3 acupuncture sessions, the patient returned to the clinic for resolution of ankle pain, ability to ambulate with weight bearing, normal local skin color (Fig. 3), and normal skin temperature. The VAS score gradually decreased to 0 as the number of acupuncture treatments increased (Fig. 4).

## 3. Discussion

Venous thrombosis of the lower extremities was ruled out in this patient, who also had a history of tinea pedis. Erysipelas of the lower limbs was diagnosed based on the patient’s signs, symptoms, and history. The occurrence of erysipelas is influenced by bacterial flora, and studies have confirmed a link between interdigital microorganisms and leg erysipelas. Moreover, the recurrence of erysipelas is often associated with interdigital tinea pedis.^[3]^ In Chinese medicine, the clinical treatment of lower limb erysipelas commonly involves herbal remedies, often combined with acupuncture bloodletting and other methods; simple acupuncture treatment alone is uncommon.

The patient primarily complained of pain in the right ankle joint, accompanied by redness and swelling of the skin on the inner side of the lower leg calf. Acupuncture was used to reduce inflammation, pain, and swelling by targeting local acupuncture points, including Ashi points, ST 41 (Jiexi), KI 3 (Taixi), KI 6 (Zhaohai), SP 4 (Gongsun), SP 5 (Shangqiu), and SP 6 (Sanyinjiao). Acupuncture has been shown to effectively reduces inflammation and pain associated with arthritis by modulating the nervous and immune systems.^[4]^ Additionally, acupuncture has demonstrated a significant improvement in skin inflammation and reduction in itching.^[5]^

This study has several limitations. Our focus on a single ethnic group limits generalizability, and the lack of long-term follow-up prevents assessment of recurrence rates. The absence of microbiological testing and objective outcome measures has reduced the robustness of our findings. Future research should address these limitations through more diverse patient populations, extended follow-up periods, and the incorporation of microbiological assessments.

## 4. Summary and conclusion

Following acupuncture treatment, the patient’s pain and local redness disappeared, suggesting that acupuncture may be effective in treating erysipelas of the lower limbs. However, owing to the limitations of this single, uncontrolled case study, definitive conclusions cannot be drawn. Further research into the use of acupuncture for treating lower limb erysipelas is warranted in the future. Additionally, it is recommended that the patient continue standard treatment for tinea pedis to prevent the recurrence of lower limb erysipelas.

## Acknowledgments

The authors would like to thank the patients for granting permission to publish their information.

## Author contributions

**Writing – original draft:** Bangwei Li.

**Writing – review & editing:** Xuanxuan Ren.

## References

[1] Ang L Ni W Lingzhi G. Risk factors of recurrent erysipelas in adult chinese patients: a prospective cohort study. BMC Infect Dis. 2021;21:26. 33413190 10.1186/s12879-020-05710-3 PMC7792156

[2] Gao LL Ye MX Sun Y Huang S Chen YJ. Clinical study of auricular acupoint therapy combined with external application of detoxification powder in the treatment of lower limb dengtuatoxin. J Pract Chin Med. 2022;36:132–4.

[3] Muller DP Hoffmann R Welzel J. Microorganisms of the toe web and their importance for erysipelas of the leg. J Dtsch Dermatol Ges. 2014;12:691–6. 10.1111/ddg.12374 24961650

[4] Yu WL Kim SN. The effect of acupuncture on pain and swelling of arthritis animal models: a systematic review and meta-analysis. Front Genet. 2023;14:1153980. 37113994 10.3389/fgene.2023.1153980 PMC10126438

[5] Park HJ Ahn S Lee H Hahm D-H Kim K Yeom M. Acupuncture ameliorates not only atopic dermatitis-like skin inflammation but also acute and chronic serotonergic itch possibly through blockade of 5-ht(2) and 5-ht(7) receptors in mice. Brain Behav Immun. 2021;93:399–408. 33524554 10.1016/j.bbi.2021.01.027

